# Characterization of a promiscuous cadmium and arsenic resistance mechanism in *Thermus thermophilus* HB27 and potential application of a novel bioreporter system

**DOI:** 10.1186/s12934-018-0918-7

**Published:** 2018-05-18

**Authors:** Immacolata Antonucci, Giovanni Gallo, Danila Limauro, Patrizia Contursi, Ana Luisa Ribeiro, Alba Blesa, José Berenguer, Simonetta Bartolucci, Gabriella Fiorentino

**Affiliations:** 1 Dipartimento di Biologia, Università degli Studi di Napoli Federico II, Complesso Universitario Monte S. Angelo, Naples, Italy; 2 Centro de Biología Molecular Severo Ochoa (CSIC-UAM), Campus Universidad Autónoma de Madrid, 28049 Madrid, Spain

**Keywords:** Thermophiles, Cadmium and arsenic resistance, Thermophilic reporter systems

## Abstract

**Background:**

The characterization of the molecular determinants of metal resistance has potential biotechnological application in biosensing and bioremediation. In this context, the bacterium *Thermus thermophilus* HB27 is a metal tolerant thermophile containing a set of genes involved in arsenic resistance which, differently from other microbes, are not organized into a single operon. They encode the proteins: arsenate reductase, *Tt*ArsC, arsenic efflux membrane transporter, *Tt*ArsX, and transcriptional repressor, *Tt*SmtB.

**Results:**

In this work we show that the arsenic efflux protein *Tt*ArsX and the arsenic responsive transcriptional repressor *Tt*SmtB are required to provide resistance to cadmium. We analyzed the sensitivity to Cd(II) of mutants lacking *Tt*ArsX, finding that they are more sensitive to this metal than the wild type strain. In addition, using promoter probe reporter plasmids, we show that the transcription of *TtarsX* is also stimulated by the presence of Cd(II) in a *Tt*SmtB-dependent way. Actually, a regulatory circuit composed of *Tt*SmtB and a reporter gene expressed from the *TtarsX* promoter responds to variation in Cd(II), As(III) and As(V) concentrations.

**Conclusions:**

Our results demonstrate that the system composed by *Tt*SmtB and *Tt*ArsX is responsible for both the arsenic and cadmium resistance in *T. thermophilus*. The data also support the use of *T. thermophilus* as a suitable chassis for the design and development of As-Cd biosensors.

**Electronic supplementary material:**

The online version of this article (10.1186/s12934-018-0918-7) contains supplementary material, which is available to authorized users.

## Background

Toxic metals and metalloids such as cadmium (Cd) and arsenic (As) are widespread environmental contaminants that pose risks to human health [1]. Microorganisms are endowed with multiple molecular mechanisms to handle exposure to these toxic compounds. In general, microbial resistance is achieved through three main mechanisms: transformation of the metals through reduction to a different oxidation state, efflux outside the cell by transporters, and/or sequestration/biosorption [2]. Common reduction mechanisms include for example the conversion of Hg^2+^ to Hg^0^, AsO_4_^3−^ to AsO_3_^3−^. Facilitated efflux transporters fall into two wide, functionally and evolutionary distinct membrane protein families, the P-type ATPases and the Major Facilitator Superfamily (MFS) of transporters [3–5]. P-type ATPases use ATP hydrolysis to transport ions across cellular membranes and are composed of three conserved domains: (1) the transmembrane (TM) helix bundle, allowing substrate translocation; (2) the soluble ATP binding domain (ATPBD) that contains the transiently phosphorylated Asp residue; (3) the soluble actuator domain (AD) [6]. Over the years, on the basis of sequence similarity and overall architecture, they have been divided into different classes: those belonging to P_1B_-type are capable to drive the efflux out of cells of both essential transition metal ions (e.g., Zn^2+^, Cu^+^, and Co^2+^) and toxic metal ions (e.g., Ag^+^, Cd^2+^, Pb^2+^) contributing to their homeostasis maintenance. A recent study on a huge number of P_1B_-type ATPase sequences combined with available biochemical data classifies them into seven distinct subfamilies (1_B-1_ 1_B-7_) on the basis of conserved motifs in TM4, TM5 and TM6, but the molecular basis of metal ion specificity remains unclear [7]. All metal efflux transporters characterized so far are tightly controlled at transcriptional level by metalloregulatory proteins which bind DNA sequences and dissociate following metal binding, thus ensuring derepression of genes encoding efflux proteins [8]. Several regulatory systems dedicated to metal/metalloid sensing have also been characterized; for instance, transcription factors of the ArsR/SmtB family are small dimeric proteins with a winged helix-turn-helix DNA binding domain controlling gene expression in response to divalent metals (e.g. zinc, cadmium) as well as metalloids (e.g. arsenic and antimonite) through an allosteric switching mechanism [9]. The exploration of life in extreme environments has led to the isolation of many thermophilic microorganisms occupying diverse extreme habitats like hot hydrothermal fluids containing high concentrations of toxic metals. For this reason they are able to cope with toxic metals, which are more soluble at high temperatures, or even to use them for their metabolism [10, 11] and are currently exploited in some bioprocesses such as biomining and bioremediation [12]. A detailed understanding of the molecular mechanisms responsible for resistance to toxic metals is also crucial for engineering organisms to develop sensitive biosensors for the detection of chemicals in the environment and to enhance bioremediation strategies [13, 14].

A significant number of whole-cell arsenic and/or cadmium biosensors has been already described in literature and is based on the realization of reporter systems containing regulatory cis-acting sequences interacting with a transcriptional repressor belonging to the ArsR/SmtB family [15, 16]. Biosensors are not intended to fully replace chemical methods but have the advantage of lower fabrication cost and higher stability and can offer on-site monitoring of even trace levels of targeted compounds in comparison with non-portable analytical methodologies [17]. To date, the major challenges in biosensor development regard the screening or modification of efficient regulator protein/promoter pair for increased sensitivity and specificity [18, 19] as well as biosensor stability over time and simultaneous monitoring of multiple environmental parameters [20].

*Thermus thermophilus* HB27 is a thermophilic aerobic Gram-negative bacterium capable of growing in the presence of arsenic concentrations that are lethal for other microorganisms [21]. In recent studies we demonstrated that the arsenic resistance system is not clustered in a single *ars* operon as in other organisms, but the genes are spread in the genome: *TTC1502* encoding a cytoplasmic arsenate reductase (*Tt*ArsC) able to reduce arsenate to arsenite, *TTC0354*, encoding a P_1B_-type membrane ATPase responsible for the efflux (herein named *Tt*ArsX) and *TTC0353* encoding a repressor (*Tt*SmtB) sensitive to both As(V) and As(III) [22–24]. *Tt*SmtB is a member of the ArsR/SmtB family, sharing 50% identity with the well characterized SmtB of *Synechococcus* PCC7942 [25]. It is a dimeric protein containing three Cys residues in a reduced state and a conserved metal binding box presumably involved in As(V) and As(III) binding. The protein can bind to a consensus regulatory sequence located upstream of *TtarsX* preferentially in an un-metallated state and in vivo *Tt*SmtB regulates *TtarsX* transcription upon arsenic interaction through a derepression mechanism [24].

In the present study, we evaluated the contribution of *Tt*SmtB and *Tt*ArsX in cadmium detoxification using a combination of genetic and physiological approaches. The results obtained support that in *T. thermophilus* the mechanism employed for survival to cadmium and arsenic exposure is promiscuous, suggesting that the evolution of shared/common defense mechanisms represents an adaptation strategy to cope with toxic metals at high temperatures whereas keeping a reduced genome. In addition, to analyze the cadmium/arsenic response of *TtarsX* promoter in dependency on varying *Tt*SmtB concentration, we settled up different β-gal reporter systems with the final goal of evaluating the utilization of thermophilic molecular components and thermostable chassis cells in biosensing applications.

## Methods

### Culture conditions and determination of minimum inhibitory concentration

Strains, genotypes and sources are summarized in Additional file 1: Table S1.

*T. thermophilus* HB27 wild type strain, *T. thermophilus ΔsmtB::kat* and *TTC0354::pK18* mutants were grown aerobically at 70 °C in TM medium as described [24].

Minimum inhibitory concentration (MIC) was determined as the lowest concentration of cadmium that completely inhibited the visible growth of the strains after overnight incubation as indicated by the lack of turbidity. Basically, exponentially growing cultures of *T. thermophilus* HB27, *T. thermophilus ΔsmtB::kat* and *TTC0354::pK18* were diluted to 0.08 OD_600 nm_ in 24 well plates containing increasing concentrations of CdCl_2_ ranging from 0 mM to 5 mM and incubated at 70 °C  or 60 °C  for 18 h; depending on the strain tolerance, the concentration interval was narrowed in consecutive experiments; the MIC endpoint was considered as the lowest Cd(II) concentration at which there was a difference between grown and start culture lower than 0.01OD_600 nm_. The values reported are the average of two independent determinations.

### In silico analysis

BlastP analyses were performed using the Blastall (v.2.2.25) program. The predicted presence and number of TMs, in the full length *Tt*ArsX, was determined using the TMHMM 2.0 online (http://www.cbs.dtu.dk/services/TMHMM) and the TM-pred online servers (http://www.ch.embnet.org/software/TMPRED_form.html) [26, 27]. Conserved motifs in TM helices of *Tt*ArsX were determined by manual inspection referring to those identified by Smith et al. [7]. The presence of Metal Binding Domain(s) (MBD) was determined by manually looking at a CXXC motif in the protein sequence and fold prediction performed at (http://pfam.sanger.ac.uk).

Models of the MBD were generated using I-TASSER web server [28] (https://zhanglab.ccmb.med.umich.edu/I-TASSER/) using as input the first 91 amino acids of *Tt*ArsX. The model was selected according to its similarity to available crystal structures of MBD of P_1B_-type ATPases from other species [6].

The docking calculations were obtained using Hex Protein Docking server [29] with *Tt*ArsX MBD and As(III) or Cd(II); 100 rigid body docking solutions were generated and the 10 best refined by energy minimization. The proposed model for the metal docked into the MBD is the structure with the smallest distance between the metal and cysteine [2.61 Å for As(III) or 2.35 Å for Cd(II)], after an energy minimization step.

### Bioreporter constructions

The regulatory region upstream of *TtarsX* (*TtarsXp*) previously named *TTC0354p*, was amplified by PCR using the primer pairs *new 0354pr fwEcoRI* and *R0354NdeI*, respectively (Additional file 1: Table S2); the region extends from − 73 to + 1 from the transcription start site [24]. The primers introduced *Eco*RI and *Nde*I restriction sites, so that the amplified fragment could be cloned in the adapted pMHbgaA plasmid [30]. The new vector was named pMH*TtarsXp*bgaA.

To obtain the plasmid pMH*TtarsXp*bgaA-nqo*Tt*smtB, the *pnqo*-*TtsmtB* gene cassette, where *TtsmtB* is under the control of the *nqo* promoter, was cloned into pMH*TtarsXp*bgaA vector; in *T. thermophilus*, the *nqo* promoter drives the expression of the operon encoding the major respiratory complex I during aerobic growth [31]. In particular, pET28*/TtsmtB* was digested with *Nde*I *Hind*III [24] and cloned into the corresponding sites of pMKpnqo-bgaA [31] giving pMKpnqo-*TtsmtB*; afterwards, the plasmid was digested with *Xba*I *Hind*III and the gel purified *pnqo*-*TtsmtB* cassette subjected to a fill-in reaction and cloned into the filled-in *Hind*III site of pMH*TtarsXp*bgaA.

pMH*TtarsXp*bgaA-nqo*Tt*SmtB was used to transform *T. thermophilus* HB27 and *TTC0354::pK18* mutants in the conditions described [24]. The pMHPnorbgaA vector was also used to transform the same strains and used as negative control [32]. Cells were then incubated for 24–48 h at 60 °C on TM plates containing hygromycin (100 μg/mL). All the plasmids used in this study are described in Additional file 1: Table S3.

### β-galactosidase assays

For measuring reporter β-galactosidase activity, the growing transformants were diluted to 0.1 OD_600 nm_ in TM medium in the presence of hygromycin (100 μg/ml), treated with different concentrations of NaAsO_2_, KH_2_AsO_4_ and CdCl_2_ (Sigma) as source of As(III), As(V) and Cd(II) respectively, and grown at 60 °C for 16 h. β-galactosidase activity assays were carried out on permeabilized cells in 96-well microplates at 70 °C with a Synergy H4 microplate reader (BioTeK) as described by Miller [33].

Miller units (U) were calculated by the equation:

$$U = \frac{{OD_{420} - (1.75 \times OD_{550} ) }}{{t({ \hbox{min} }) \times {\text{V}}({\text{mL}}) \times OD_{600} }}$$

where: OD_420_ = OD of the chromogenic product, OD_550_ = OD of the cellular debris, t = time of reaction, V = volume of used cells and OD_600_ = OD of the cell culture. β-galactosidase activity of *T. thermophilus* negative control (transformed with pMHPnorbgaA vector) was subtracted from that of the samples. The activity reported is the average of two or three independent experiments each made in triplicate. The error bars indicate the standard deviation of the average values. Miller Units expressed as a percentage, were calculated assuming that the Miller Units value of not treated cells (control) was 100%. Statistical analysis was performed using a Student’s t test; significant differences are indicated as: *p < 0.05, **p < 0.01, ***p < 0.001, ****p < 0.0001.

### Purification of *Tt*SmtB

Recombinant *Tt*SmtB was purified to homogeneity using the procedure already described, consisting of thermo-precipitation of the *E. coli* BL21-Codon Plus (DE3) RIL/*Tt*SmtB cell extract followed by HiTrap Heparin chromatography. The histidine tag was removed from purified *Tt*SmtB by thrombin digestion (Sigma). The purified protein was stored in aliquots at − 20 °C [24].

### Electrophoretic mobility shift assay (EMSA)

To determine if cadmium was ligand of *Tt*SmtB, electrophoretic mobility shift assays (EMSA) were performed. The *TtarsX* promoter region was amplified by PCR using specific primer pair: *0354footprint fw* and *0354footprint rv,* (Additional file 1: Table S2). EMSA reactions were set up as described [34], using 5 µM of proteins pre-incubated or not with Cd(II) at molar ratio of 1:20 and 1:50 (considering *Tt*SmtB as a dimer).

## Results and discussion

### Domain organization and subfamily classification of *Tt*ArsX

**Fig. 1:**
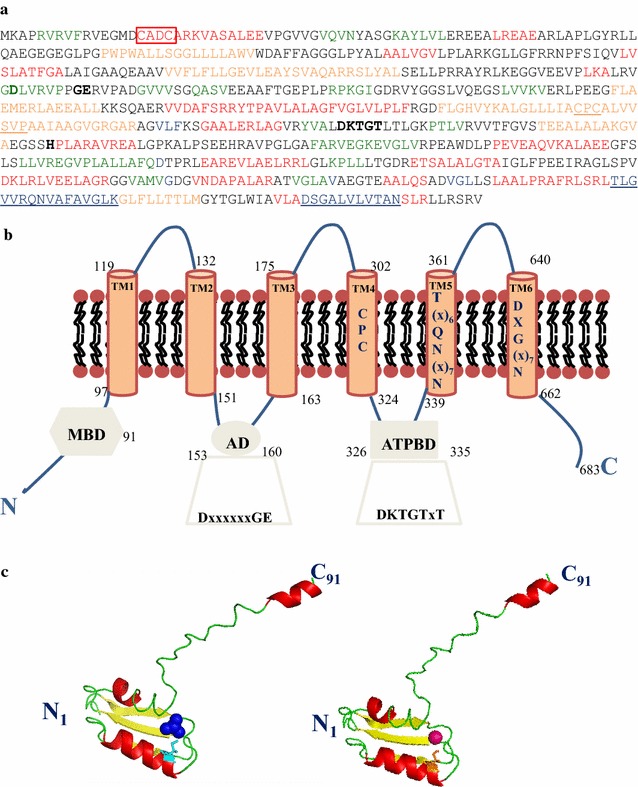
a Primary structure of *Tt*ArsX. Aminoacids that are predicted to be in β-turns are colored green, those in α-helices are red and those in turns are colored black. Aminoacids in orange are included in TM helices. The residues conserved in P-type ATPases are shown in bold, those which are signatures of the P_1b-2_ type subfamily are underlined. The CXXC motif at the N-terminus is boxed. b Schematic topology of *Tt*ArsX (adapted from [7]). MBD: soluble N-terminal metal-binding domain (hexagon); AD: actuator domain (ellipse), ATPBD: ATP binding domain. Invariant motifs in ATPBD and AD domains of P-type ATPases are shown in bold. Residues indicated in the TM helices 4,5,6 are those conserved in P_1b-2_ type subfamily. c 3D model of *Tt*ArsX MBD (corresponding to residue 1–91 from the intact protein) interacting with As(III), (colored blue, left) and Cd(II) (magenta, right)

Our recent work showed that TTC0354, herein named *Tt*ArsX, is a membrane metal-transporter involved in arsenic detoxification; in fact, a mutant strain in which this gene had been knocked out, was about 15-fold more sensitive to both As(III) and As(V) treatment than the wild type [24]. In order to analyse the putative role of this protein in the detoxification of metals different from As(III)/As(V), in particular Cd(II), at first some in silico studies were performed aimed at identifying divergence or not in conserved motifs. TM helix prediction tools suggested that *Tt*ArsX is composed of six TM helices containing all the conserved signatures of the characterized P_1B-2_-subclass members which generally display a dual role in Zn^2+^ transport and toxic metal ion detoxification, especially Cd(II) [7, 35]. They are: SXP and CPC motifs in TM4, a T(X)_5_QN(X)_7_K motif in TM5 and a DXG(X)_7_N in TM6 (Fig. 1a in bold). A previous work had already shown that *Tt*ArsX is a P_1B_-type ATPase stimulated in vitro by Zn^2+^/Cd^2+^ cations but with an unclear role in their tolerance in vivo [23]. The overall topology of *Tt*ArsX (Fig. 1b) obtained integrating results from a 3D model of the protein [24] also helped to identify a soluble MBD containing the CXXC motif that, as indicated by docking analyses, could be responsible for both Cd(II) and As(III) recognition inside the cell (Fig. 1c).

Taken together, the results of in silico analysis assign *Tt*ArsX to the well characterized P_1B-2_-subfamily, show the presence of a soluble heavy metal associated domain (Pfam: PF 00403) and suggest a wider metal ion specificity than that previously known.

### Contribution of *Tt*ArsX and *Tt*SmtB to the Cd(II) tolerance mechanism

To analyse the role of *Tt*ArsX and the transcriptional repressor *Tt*SmtB [24] in resistance to cadmium, we used different physiological and genetic approaches.

First, we compared the growth of *T. thermophilus* HB27 and two mutants defective in *Tt*ArsX or *Tt*SmtB (*T. thermophilus TTC0354::pK18* and *T. thermophilus ΔsmtB::kat*, respectively) in the presence of different Cd(II) concentrations, and determined the MIC values which are reported in Table 1. *T. thermophilus TTC0354::pK18* revealed a 15-fold increase in cadmium sensitivity in comparison to the wild type, thus showing a role of *Tt*ArsX in Cd(II) resistance; on the other hand, *T. thermophilus ΔsmtB::kat*, showed a 3-fold increase in cadmium sensitivity as compared to wild type HB27. One possible explanation for this unexpected result is a polar effect of the *TtsmtB::kat* mutation: in the mutant strain the kanamycin resistance gene is in counter sense; therefore, the basal levels of *Tt*ArsX could be lower than in the wild type, making the cells more sensitive.

**Table 1:** Bacterial resistance to cadmium and arsenic

MIC			
Strain	Cd(II) (mM)	As(III)^a^ (mM)	As(V)^a^ (mM)
*T. thermophilus* HB27	3	40	44
*T. thermophilus TTC0354::pK18*	0.2	3	3
*T. thermophilus ΔsmtB::kat*	1	32	18

MICs were determined in TM medium with increasing concentrations of cadmium (0–5 mM) as already measured for As(V) and As(III) [23^a^]

In addition, the comparison of MIC values with those previously reported for As(V) and As(III) showed that *T. thermophilus* HB27 is almost 14-fold more sensitive to Cd(II) than to arsenic (Table 1).

**Fig. 2:**
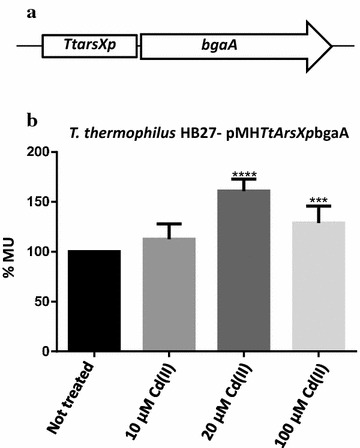
Cd(II) dependent activity of *TtarsX* promoter with transcriptional* bgaA* fusions. a Schematic representation of the reporter system used to transform *T. thermophilus* HB27 and *TTC0354::pK18*. b β-gal activity expressed in % MU of *T. thermophilus* HB27-pMH*TtarsXp*bgaA treated or not with 10, 20 and 100 µM of CdCl_2_. Statistical analysis was performed using the Student’s t test; significant differences are indicated as: *p < 0.05, **p < 0.01, ***p < 0.001, ****p < 0.0001

To analyse in vivo whether *TtarsX* promoter had Cd(II) responsive activity, the regulatory region was cloned in a promoter probe vector upstream of the *bgaA* gene, encoding a thermostable β-galactosidase [30]. The plasmid pMH*TtarsXp*bgaA (Fig. 2a) was transformed into *T. thermophilus* HB27 and the β-gal activity was measured also upon Cd(II) treatment at 10, 20 and 100 µM (see “Methods”). The results in Fig. 2b show that transcription from *TtarsX* promoter in the reporter system is activated by Cd(II) by twofold at 20 µM (633 ± 84 Miller Units, MU) in comparison to values in untreated cells (393 ± 40 MU). At 100 µM Cd(II) transcription is also increased in comparison to the control, but at lower levels; the reduced activity could be the consequence of partial toxicity, since reduced growth rates were observed under these conditions (Additional file 1: Figure S1).

**Fig. 3:**
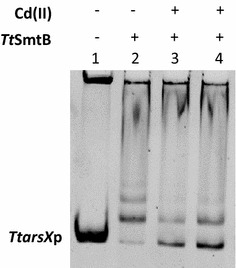
EMSA analysis of *Tt*SmtB in the presence of Cd(II). Binding of *Tt*SmtB to *TtarsXp* without (lane 2) and with CdCl_2_ at molar ratio of 1:20 and 1:50 (lanes 3–4)

To check whether *Tt*SmtB was the transcriptional regulator of *Tt*ArsX in the presence of Cd(II), we performed in vivo and in vitro experiments. The comparison of β-gal activities in *T. thermophilus* HB27-pMH*TtarsXp*bgaA and *T. thermophilus* *ΔsmtB::kat*-pMH*TtarsXp*bgaA grown in the absence of metals (393 ± 40 and 887 ± 138 MU, respectively), is consistent with the in vivo role of *Tt*SmtB as a repressor. Moreover, EMSA assays were carried out with recombinant *Tt*SmtB on the *TtarsX *promoter after preincubation with or without Cd(II). As shown in Fig. 3, our results confirm that the protein interacts with *TtarsXp* and indicate that, upon binding with Cd(II), binding of *Tt*SmtB to the promoter is hampered. *Tt*SmtB DNA-binding behavior upon interaction with Cd(II) is similar to that already observed with As(III) and As(V) [24], suggesting that metal ions could take contacts with the same protein motif. Moreover, these results strongly support the role of *Tt*SmtB in Cd(II) mediated *TtarsX* transcription where it works as repressor.

Altogether, these results indicate that *Tt*SmtB regulates cadmium tolerance by controlling at transcriptional level the metal efflux gene, adopting a derepression mechanism similar to that employed for arsenic detoxification [24].

### Bioreporter construction and characterization

**Fig. 4:**
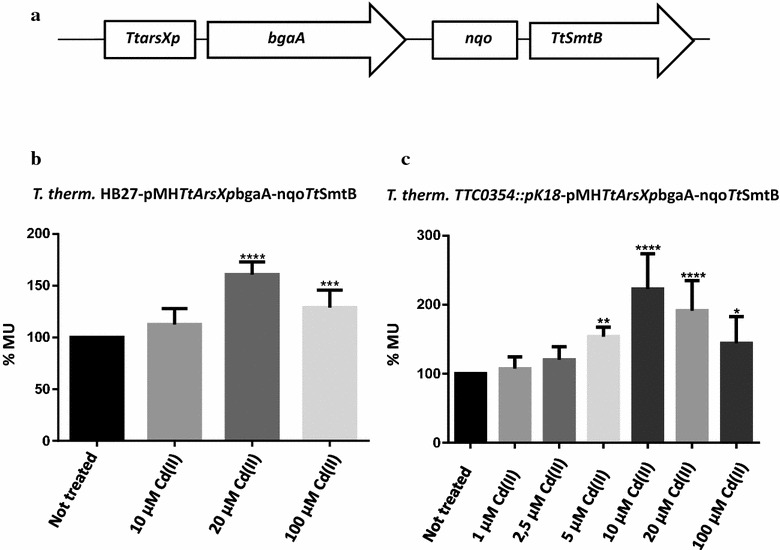
Cd(II) dependent bioreporter response. a Schematic representation of the recombinant vector used to transform *T. thermophilus *HB27 and *TTC0354::pK18*. b β-gal activity expressed in % MU of *T. thermophilus *HB27-pMH*TtarsXp*bgaA-nqo*Tt*SmtB treated or not with 10, 20 and 100 µM of CdCl_2_. c β-gal activity expressed in % MU of *T. thermophilus* *TTC0354::pK18*-pMH*TtarsXp*bgaA-nqo*Tt*SmtB treated or not with 10, 20 and 100 µM of cadmium. Statistical analysis was performed using a Student’s t test; significant differences are indicated as: *p < 0.05, **p < 0.01, ***p < 0.001, ****p < 0.0001

To evaluate the potential of the pair composed by *Tt*SmtB and *TtarsX* responsive promoter as components of a bioreporter system for toxic metal detection, we aimed at characterizing their cadmium and arsenic sensing potential in engineered *T. thermophilus* cells. Hence, we generated a new reporter plasmid, pMH*TtarsXp*bgaA-nqo*Tt*SmtB, to measure the transcription of the reporter gene from the *TtarsX* promoter in a context in which the *Tt*smtB gene was expressed constitutively from the nqo promoter (Fig. 4a). We expected that an increase in the intracellular concentration of the transcription factor allowed a more efficient repression of the system and, as a consequence, an activity of the reporter gene mainly depending on metal concentration. As shown in Fig. 4b, the system responds to increasing concentrations of Cd(II) almost in a linear form in a wide range, but with a slope too flat to measure any concentration accurately. As we suspected that this was due to the presence of *Tt*ArsX actively extruding Cd(II), we assayed the bioreporter in *T. thermophilus* *TTC0354::pK18* defective mutant. As expected (Fig. 4c), the increase in bioreporter activity was higher in the mutant strain than in the parental one; in fact at 10 µM Cd(II) a twofold induction was measured in the first, whereas no significant induction occurs in the wild type; furthermore, the mutant strain was able to detect Cd(II) at 5 µM, a concentration value that is fourfold lower than that required to get a similar signal in the parental strain. However, the tradeoff of this system is that in the mutant strain the response decreases above 20 µM Cd(II). Therefore, it could be envisioned to use both host systems to cope with a wider range of Cd(II) concentrations. The increase in the sensitivity of a biosensor using strains with mutations in the arsenic efflux pump in place of the wild type has been also reported for *P. putida* [36].

**Fig. 5:**
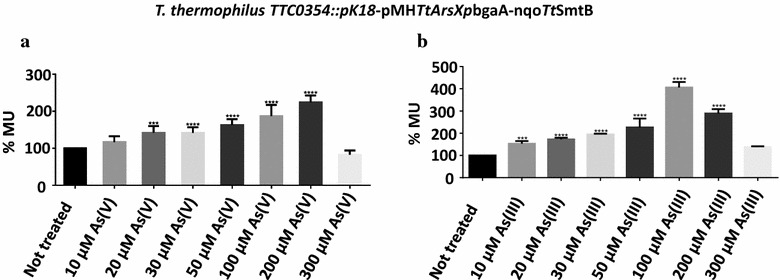
As(V) and As(III) dependent bioreporter response. a β-gal activity expressed in % MU of *TTC0354::pK18*-pMH*TtarsXp*bgaA-nqo*Tt*SmtB treated or not with 10, 20, 30, 50, 100, 200 and 300 µM of As(V). b β-gal activity expressed in % MU. of *T. thermophilus TTC0354::pK18*-pMH*TtarsXp*bgaA-nqo*Tt*SmtB treated or not with 10, 20, 30, 50, 100, 200 and 300 µM of As(III). Statistical analysis was performed using a Student’s t test; significant differences are indicated as: *p < 0.05, **p < 0.01, ***p < 0.001, ****p < 0.0001

As previous functional studies showed that *Tt*SmtB and *Tt*ArsX are part of the arsenic resistance system, we evaluated the bioreporter system for its capability to detect arsenate and arsenite in *T. thermophilus* *TTC0354::pK18*. Figure 5 shows that the first significant reporter signal was obtained at 20 µM As(V) and 10 µM As(III), demonstrating that inactivation of Cd/As efflux pump translates in higher response sensitivities to arsenic too. As also observed for Cd(II), the reporter activity increases to a maximum level [385 ± 22 MU for As(V) and 563 ± 65 MU for As(III)] after which it decreases likely because of the metal toxicity. Overall, this study underlines the advantage of using: (i) an extremophilic microorganism more resistant to stress conditions for the development of biosensor for in situ monitoring of contaminated sites [37]; (ii) a bioreporter system where the interaction between the regulator and its target promoter has been analyzed and defined [38]; (iii) a strain genetically modified more sensitive to detect metals [36]. In addition, these results encourage to improve the newly developed cell-based system for the realization of a robust biosensor for multiple metal detection [39]. In this context, a system to be used as a screening tool to detect metals in environmental samples could significantly help to find metal polluted areas, or to monitor in situ cleanup during bioremediation.

## Conclusions

This work reports for the first time the identification of a molecular mechanism responsible for cadmium tolerance of the thermophilic bacterium *T. thermophilus*, taking advantage of the utilization of suitable genetic tools. Interestingly, the system includes part of the machinery (*Tt*ArsX and *Tt*SmtB, membrane efflux ATPase and ArsR/SmtB transcriptional repressor, respectively) used to cope with arsenic; to the best of our knowledge this is the first functional characterization in a bacterium of a common/promiscuous mechanism to defend from both arsenic and cadmium, giving new venues for the understanding of the metal response evolution and the adaptation of environmental thermophilic microorganisms to deal with high concentrations of metals under enhanced solubilization conditions [12]. Notably, from an evolutionary point of view it can be speculated that in the absence of an *ars* operon, cells may have evolved arsenic resistance from preexisting metal detoxification systems. It is also plausible that promiscuous detoxification systems have developed according to the hypothesis that in the genomes of thermophilic microorganisms the genetic information is condensed. Moving to biotechnologically relevant applications in the improvement of biosensor field, this study outlines the importance of a detailed characterization of the molecular components (intrinsic promoter activity, repressor/promoter and repressor/metal(s) binding affinities) and points to *T. thermophilus* as suitable chassis cell for design and development of robust metal biosensors. In this context, advantageous modifications can be programmed to increase biosensor sensitivity, selectivity and/or ability to detect metal mixtures.

## Additional file

Additional file 1: Table S1. Strains used in this work classified according to their genotype. Table S2. Oligonucleotides used in this work. Table S3. Plasmids used in this work classified according to their features. Figure S1. Growth curves of *T. thermophilus* HB27 transformed with the vector pMH*TtarsXp*bgaA in the absence (circle) and presence of 100 μM Cd(II) (triangle).
