# Supplementary material for: Characterization of a promiscuous cadmium and arsenic resistance mechanism in Thermus thermophilus HB27 and potential application of a novel bioreporter system
Source: Microb Cell Fact. 2018 May 18;17:78. doi: 10.1186/s12934-018-0918-7 (PMC5960188; doi:10.1186/s12934-018-0918-7)
Supplement: Supplementary file 1 — Additional file 1: Table S1. Strains used in this work classified according to their genotype. Table S2. Oligonucleotides used in this work. Table S3. Plasmids used in this work classified according to their features. Figure S1. Growth curves of T. thermophilus HB27 transformed with the vector pMHTtarsXpbgaA in the absence (circle) and presence of 100 μM Cd(II) (triangle). [file 12934_2018_918_MOESM1_ESM.docx]

**Table S1.** Strains used in this work classified according to their genotype.

| **Strain** | **Genotype** | **Source** |
| --- | --- | --- |
| *T. thermophilus* HB27 | Wild type | DSMZ |
| *T. thermophilus* *ΔsmtB*::*kat* | *T. thermophilus* HB27 deletion mutant of the *TtsmtB* gene, Kan^r^ | [23] |
| *T. thermophilus* *TTC0354::pK18* | *T. thermophilus* HB27 insertion mutant defective in *TTC0354,* Kan^r^ | [23] |
| *E. coli* BL21-Codon Plus (DE3) RIL - *Tt*SmtB | F^–^ ompT hsdS(r_B_– m_B_–) dcm+ Tet^r^ gal λ (DE3) endA Hte [argU ileY leuW Cam^r^] | [23] |

**Table S2.** Oligonucleotides used in this work.

| **Primer names** | **Sequences** (the restriction sites are underlined) 5’-3’ |
| --- | --- |
| *0354pfwEcoRI* | AAAGAATTCCTGTTGGCGGAGGCCCTG |
| *0354prv0354NdeI* | AGCCTTCATATGCCCAGGGTAGC |
| *0354footprint fw* | CGCCTCGCCGACCGGCA |
| *0354footprint rv* | CCATGCCCTCTACCCGGAAG |

**Table S3.** Plasmids used in this work classified according to their features.

| **Plasmid** | **Key features** | **Source** |
| --- | --- | --- |
| pMHbgaA | β-galactosidase, hygromycin B resistance. | [25] |
| pMH*TtarsX*pbgaA | *TtarsX* promoter, β-galactosidase, hygromycin B resistance. | This work |
| pMKpnqo-bgaA | *nqo* promoter, β-galactosidase, kanamycin resistance. | [26] |
| pMKpnqo-*TtsmtB* | *nqo* promoter, *Tt*SmtB, kanamycin resistance | This work |
| pMH*TtarsX*pbgaA-nqo*Tt*SmtB | *TtarsX* promoter, β-galactosidase, *nqo* promoter, *Tt*SmtB, hygromycin B resistance | This work |
| pMHPnorbgaA | *nor* promoter, β-galactosidase, hygromycin B resistance | [27] |
| pET28b(+)/*Tt*SmtB | T7lac , *lac*I repressor, *Tt*SmtB , kanamycin resistance | [23] |

**Figure S1.**


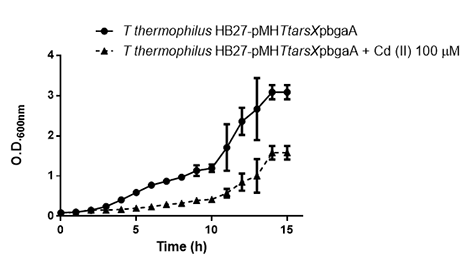


**Fig. S1** Growth curves of *T. thermophilus* HB27 transformed with the vector pMH*TtarsX*pbgaA in the absence (circle) and presence of 100 μM Cd(II) (triangle).
